# Removal of Different Dye Solutions: A Comparison Study Using a Polyamide NF Membrane

**DOI:** 10.3390/membranes10120408

**Published:** 2020-12-10

**Authors:** Asunción María Hidalgo, Gerardo León, María Gómez, María Dolores Murcia, Elisa Gómez, José Antonio Macario

**Affiliations:** 1 Departamento de Ingeniería Química, Facultad de Química, Campus de Espinardo, Universidad de Murcia, 30100 Murcia, Spain; maria.gomez@um.es (M.G.); md.murcia@um.es (M.D.M.); egomez@um.es (E.G.); joseantonio.macario@um.es (J.A.M.); 2 Departamento de Ingeniería Química y Ambiental, Universidad Politécnica de Cartagena, 30202 Cartagena, Spain; gerardo.leon@upct.es

**Keywords:** characterization, dyes, molecular structure, nanofiltration, physico-chemical properties

## Abstract

The removal of organic dyes in aquatic media is, nowadays, a very pressing environmental problem. These dyes usually come from industries, such as textiles, food, and pharmaceuticals, among others, and their harm is produced by preventing the penetration of solar radiation in the aquatic medium, which leads to a great reduction in the process of photosynthesis, therefore damaging the aquatic ecosystems. The feasibility of implementing a process of nanofiltration in the purification treatment of an aqueous stream with small size dyes has been studied. Six dyes were chosen: Acid Brown-83, Allura Red, Basic Fuchsin, Crystal Violet, Methyl Orange and Sunset Yellow, with similar molecular volume (from 250 to 380 Å). The nanofiltration membrane NF99 was selected. Five of these molecules with different sizes, shapes and charges were employed in order to study the behavior of the membrane for two system characteristic parameters: permeate flux and rejection coefficient. Furthermore, a microscopy study and a behavior analysis of the membrane were carried out after using the largest molecule. Finally, the Spiegler–Kedem–Katchalsky model was applied to simulate the behavior of the membrane on the elimination of this group of dyes.

## 1. Introduction

Organic dyes (such as Acid Brown-83, Allura Red, Basic Fuchsin, Crystal Violet, Methyl Orange and Sunset Yellow) can be found in effluents of different industries (food, medical, painting) but the most pollutant industry is the textile.

The discharge of these pollutants into the aquatic environment has a strong environmental impact due to the amount of toxic compounds they have and also due to the fact that they cause a decrease in the self-purification capacity of the water they are discharged into. This phenomenon prevents plants from performing photosynthesis and microorganisms from developing their biological activity [1].

Therefore, there are numerous methods of disposal of dye aqueous solutions, which can be grouped into physical, chemical and biological methods, but none of them stand out among the others [2,3,4,5]. Following this, new techniques are being investigated, including membrane technologies, because they offer low costs and give good yields [1,6].

As a result, membrane technology is attracting great interest. This technology is based on the separation of compounds by size and charge, as the membrane acts as a filter that retains the molecules which are larger than the pore and allows the water to pass through. In the last decade, more than 65% of research works have been based on the fabrication strategies of nanoporous membranes and their applications in the field of water purification [7,8,9,10,11,12]. According to Wang et al. the solute and water permeability play important roles in the membrane performance. The membrane is able to separate pollutants from water mainly through size exclusion and solute diffusion [7].

The application of pressure driven membrane processes for the removal of low molecular weight organic compounds from aqueous solutions has been described in several recent publications—for example, phenol and chlorophenol compounds [13]. A comparative study, using different organic compounds (atrazine, aniline and phenol, and their derivatives 4-chlorophenol, 4-nitrophenol and 4-nitroaniline) in aqueous solution and their elimination through NF-97 polyamide membrane, was carried out. The different physicochemical parameters of the organic compounds, the permeate flux and the rejection coefficient values were found to be correlated. The best correlation for the rejection coefficient was obtained using the molar refractivity and the water solubility of the compound simultaneously. For permeate flux, the best correlation value was obtained using the surface tension and molecular weight [14].

It is clear that removal efficiency depends on the membrane type and solute, and the interaction between them. Temperature, pH, pressure and concentration also influence rejection [15]. Whether nanofiltration should be used in the treatment of wastewater containing dyes depends on the rejection capacity of the membranes and the permeate flux.

In addition, distilled water tests were performed in order to characterize the membrane, and selectivity tests facing salt solutions before and after dyes tests were carried out in order to know the membrane permeability, studying performance and its changes during the process. In that way, membrane fouling can be analyzed, as well as the phenomenon in which membrane pores get wider because substances passing, known as swelling, can be observed [16,17,18].

The discussion on membrane-based treatment processes is incomplete without an elaborate perception of the mechanism governing the transport of solute across the membrane and compressive modeling of a membrane-based technique [1].

The main goal of this research work is to study the behavior of the NF99 membrane on the elimination of several dyes, which are molecules of different structure, charge and shape, the following ones being chosen: Allura Red, Basic Fuchsin, Crystal Violet, Methyl Orange and Sunset Yellow. These molecules were selected since in the bibliography there are no studies for some of them, such as Basic Fuchsin and Allura Red. Solutions of each dye were used to characterize the system and to obtain the values of the permeate flux and rejection coefficient. Furthermore, a preliminary study on the characterization of the membrane treated with salt solutions was carried out before and after the dye treatment. Such a study was complemented by scanning electric microscopy (SEM) morphologic study of the membrane using the Acid Brown-83 dye. This molecule was selected because it is a real case of a leather tanning industry located in Murcia (Spain). Finally, the Spiegler–Kedem–Katchalsky model was applied to simulate the behavior of the membrane on the elimination of this group of dyes.

## 2. Materials and Methods

### 2.1. Materials

#### 2.1.1. Membrane

A nanofiltration membrane was employed in this research. Its main technical characteristics are shown in Table 1.

#### 2.1.2. Reagents

The following reagents were used in the assays:

- Acid Brown-83 (AB83), C_18_H_13_N_6_NaO_8_S and its molecular weight is 496.39 g/mol. Supplied by Alfa Industries (Spain).
- Allura Red (AR), C_18_H_14_N_2_Na_2_O_8_S_2_. Its molecular weight is 496.44 g/mol, 80% of purity. Supplied by Sigma-Aldrich INC (Germany).
- Basic Fuchsin (BF), C_20_H_20_ClN_3_. Its molecular weight is 337.86 g/mol. Supplied by Sigma-Aldrich (Germany).
- Crystal Violet (CV), C_20_H_11_N_2_Na_3_O_10_S_3_. Its molecular weight is 407.98 g/mol and ≥90% of purity. Supplied by Sigma-Aldrich INC (Germany).
- Methyl Orange (MO), C_14_H_14_N_3_O_3_NaS. Its molecular weight is 327.33 g/mol. Supplied by Probus (Spain).
- Sunset Yellow (SY), C_16_H_10_N_2_Na_2_O_7_S_2_. Its molecular weight is 452.37 g/mol and 80% of purity. Supplied by Sigma (Germany).
- Sodium Chloride (NaCl). Its molecular weight is 58.4 g/mol. Supplied by Panreac (Spain).
- Hydrous magnesium chloride, MgCl·6H_2_O. Its molecular weight is 203.30 g/mol. Supplied by Panreac (Spain).

In Table 2, Log K_wo_, pK_a_ and water solubility data, obtained using PubChem, are shown.

### 2.2. Equipment

The research was carried out in a membrane module from INDEVEN CF (Spain), which has been designed at laboratory scale to obtain further information on the behavior of plane membranes with small surface area. In addition to the membrane module, other equipment was used to obtain valuable parameters for further comparison and discussion among the different dyes.

#### 2.2.1. Membrane Module

The membrane module consists of three main stages of installation: feed tank, fluid impulsion pump and membrane settlement. Furthermore, there is a manometer and a rotameter that measure rejection pressure.

The feed tank is cylindric and it maintains the internal fluid at a constant temperature. Its capacity is of 12 L. The fluid passes from the feed tank to the driving pump through a flexible rubber pipe. The pump is a triple plunger pump from Flowmax (Spain). It consists of three AISI 316 Steel valves and of corrosion resistant double collectors. Flow rate is controlled by a manual needle valve.

The membrane inflows are divided into two: permeate flow and concentrate flow. The last one re-enters the feed tank. Moreover, the vent plug discharge and the caudal control are carried out by a flow that leaves the impulsion pump and arrives to the feed tank.

The membrane, whose surface is 30 cm^2^, is placed near the feeding spacer, with the active layer looking towards the mainboard. The following step involves placing the permeate spacer and finally the closing plate. Two o-rings seal the set.

Continuous functioning is guaranteed because the concentrate flow discharges in the feed tank. Operating pressure is regulated by a valve and a manometer, and the flow is measured by a rotameter from TechFluid, which detects flows ranging from 50 to 400 L/h.

#### 2.2.2. Spectrophotometer

A spectrophotometer from Shimadzu (UV–160) (Japan) was employed to measure the absorbance of the dye samples. The measurements were carried out at specific wavelengths, which were 443 nm for Acid Brown-83, 485 nm for Sunset Yellow, 596 nm for Crystal Violet, 500 nm for Allura Red, 460 nm for Methyl Orange and 550 nm for Basic Fuchsin.

#### 2.2.3. Variable Pressure Scanning Electron Microscope

To develop the membrane fouling study, a scanning electron microscope from Hitachi (S-3500N model) (Japan) was employed; its main characteristics are the following:

- Resolution: 3 nm (high vacuum mode) or 4.5 nm (low vacuum mode);
- Zoom: 15–300,000;
- Accelerating voltage: 0.3–30 kv;
- Variable pressure range: 1–270 Pa;
- Secondary electrons detector;
- Robinson’s backscattered electron detector;
- Secondary electrons in variable pressure detector;
- X-ray detector;
- Eucentric plate with computer control and motorized movements in X, Y, Z, R and T;
- Crio-SEM cooling plate (−190 ± 60 °C);
- Peltier’s cooling sample holder (−15 ± 50 °C).

### 2.3. Experimental Series

In order to obtain further knowledge about the membrane behavior, a series of experiments with different dyes were carried out. In these series of experiments, all the operating conditions remained unchanged excluding that which was subject of study.

#### 2.3.1. Distilled Water Assays

The tank was filled with distilled water and afterwards a series of experiments were carried out. The experiments were of 15 min of length, at 5, 10 and 15 bar operating pressures and with a 150 L/h flow. The main goal of these assays was to get to know the permeability of the membrane.

#### 2.3.2. Salts Assays

The objective of the experimental assays using salt solutions was to obtain the membrane rejection coefficient and, as a result, its selectivity. In the same way that distilled water assays, the experiments were carried out at 5, 10 and 15 bar operating pressure and with a 150 L/h flow; however, the duration time was of 20 min. Aqueous solutions of 1 g/L were used to carry out the experimental assays. The salts employed in the experiments were magnesium chloride and sodium chloride.

#### 2.3.3. Dyes Assays

In order to elucidate the membrane elimination power in detail, a 50 mg/L dye dissolution was employed to fill the feed tank and 30 min assays were carried out, in which the samples were taken every 5 min with different operating pressures. Duplicate assays were carried out.

- Firstly, while the pH was maintained constant at 7 and the flow at 150 L/h, the operating pressure was varied: 10 and 15 bar.
- With the aim of finding out the influence of the pH, the previous experimental series were repeated, changing the pH: first at 8 and afterwards at 3.
- Finally, once all of these assays were carried out, the distilled water assay and the salts assay were repeated to check if the membrane had lost permeability after its use.

## 3. Results and Discussion

### 3.1. Membrane Characterization

The initial membrane characterization was carried out by determining its permeability coefficient, its performance regarding flows and its selectivity against two different salt solutions: sodium chloride and magnesium chloride.

#### Permeability Coefficient Determination

In order to determine the permeability coefficient, the following equation was used:

(1) $$J_{v}=L_{p}\cdot (\Delta P-\Delta \Pi )$$

The osmotic pressure gradient can be ignored only if the solvent is employed alone. As a result, the previous equation can be described as:

(2) $$J_{v}=L_{p}\cdot \Delta P$$

The permeability coefficient value is obtained by representing the final values of the solvent mass flow against applied pressure.

In Table 3, the permeability coefficient for the solvent (*L_p_*) values obtained in this research for different pressure ranges and the permeability coefficient values found in the literature are shown. As can be observed, the values obtained in this research are of the same order as those found in the literature [19,20,21].

### 3.2. Determination of Selectivity and Performance of the Membrane against Salt Solutions

The characterization of NF membranes is often carried out using divalent salt solutions. In this research, two salt solutions were used: sodium chloride and magnesium chloride.

To determinate the selectivity of the membrane, the rejection coefficient was established:

(3) $$\%R=\frac{(C_{0}-C_{p})}{C_{0}}\cdot 100$$

The experimental values obtained for permeate flux and rejection coefficient were treated by applying the solution-diffusion model [22]. As a result, the permeability coefficient for the solute (Ps) for each salt solution was obtained.

Table 2 shows the Ps values for each salt solution assayed, which are very close to those obtained by previous authors [13].

### 3.3. Influence of the Chemical Structure of Different Dyes

Usually, parameters such as molecular weight, log Kw and pKa, were used to explain the membrane selectivity and the rejection coefficient. However, in recent years, to attempt to explain the behavior of the nanofiltration systems, based on the two characteristic parameters, the permeate flux and the rejection coefficient, the influence of chemical structure parameters could represent an important factor to consider.

**Figure 1:**
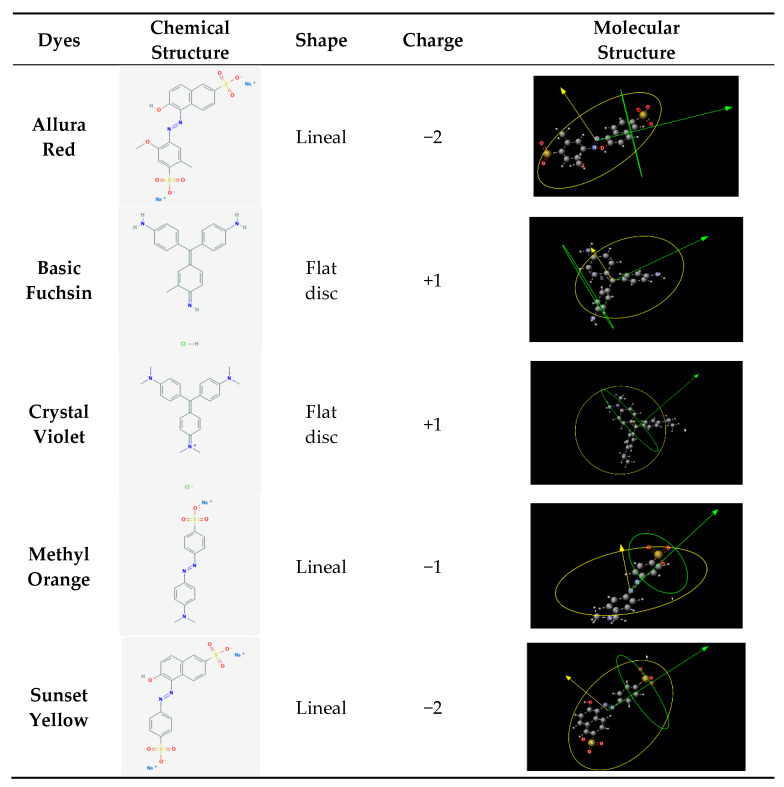
Molecular properties of the dyes.

Table 4 shows the chemical structure parameters of the dye molecules. The parameters, such as area, radio, length and volume were obtained by the program, MarvinSketch version 15.12.7, using ChemAxon. Furthermore, Figure 1 shows the charge, shape and geometry of different molecules using a tridimensional draw. These parameters were proven to influence permeate flux and rejection coefficient.

**Figure 2:**
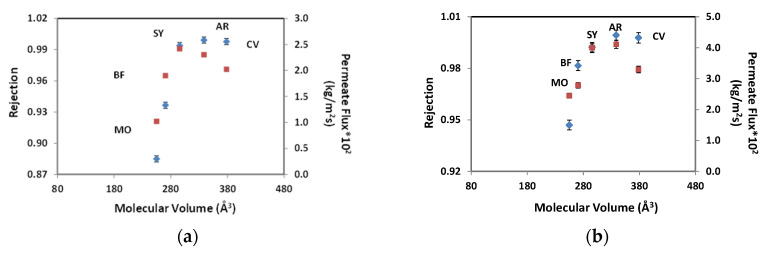
Rejection coefficient (♦) and permeate flux (■) variation with molecular volume for colorants: (MO) Methyl Orange, (BF) Basic Fuchsin, (SY) Sunset Yellow, (AR) Allure Red, (CV) Crystal Violet. Experimental conditions: pH = 7, [Dyes] = 50 mg/L and pressure values (**a**) 10 bar and (**b**) 15 bar.

In the literature, some authors have described that the most influential parameter is molecular volume, but other parameters related to chemical properties can also be used to predict the behavior of these systems. Figure 2 shows the influence of molecular volume in rejection coefficients and permeate flux using a pressure of 10 bar (a), and 15 bar (b) for the different dyes.

As can be seen in Figure 2a, when the molecular volume is increased, the selectivity of the membrane increases too. When comparing the values obtained for the different pressures applied, it is seen that high-volume molecules present a small decrease in the rejection coefficient when the pressure applied is high (15 bar). Besides that, the permeate flux has no predictive behavior because both the small dye molecules (MO and BF) that have a similar size to the molecular weight cut off MWCO of the membrane and the large dye molecules (CV) have low permeate fluxes.

According to Cheng et al. (2016), membrane water permeability and solute rejection can be attributed to sensitive pore size and membrane charge. This separation discerned three mechanisms, size exclusion (sieving), electrostatic repulsion (Donnan exclusion) and adsorption. The rejection of neutral molecules and large dye molecules (CV) was mostly size exclusion. The rejection of the low-charged solutes was dominated by the electrostatic interactions, including repulsion (cations) and attraction (anions) (BF) [15].

**Figure 3:**
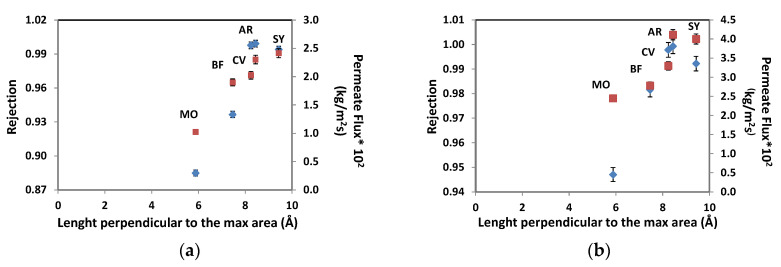
Rejection coefficient (♦) and permeate flux (■) variation with length perpendicular to the maximum area for colorants: (MO) Methyl Orange, (BF) Basic Fuchsin, (SY) Sunset Yellow, (AR) Allure Red, (CV) Crystal Violet. Experimental conditions: pH = 7, [Dyes] = 50 mg/L and pressure values (**a**) 10 bar and (**b**) 15 bar.

Furthermore, different parameters of the structure of the molecules were correlated with the permeate fluxes and rejection coefficients obtained (Figures S1–S4, Supplementary Material), and it was tested that the parameter that presents a greater incidence is length perpendicular to the maximum area. Figure 3 shows the influence of perpendicular length to the maximum area on permeate flux and rejection coefficient for (a) 10 bar and (b) 15 bar pressures. In this case, the highest value of length perpendicular to the maximum area corresponds to Sunset Yellow dye, and the values obtained for permeate flux and rejection coefficient show a lineal correlation with this parameter, in this particular range studied.

### 3.4. Influence of pH: Comparison of Electrostatic Interaction and Membrane Performance

The permeate fluxes and rejection coefficients obtained from the dye molecule assays were studied with different pH values using the NF99 membrane under identical conditions and the obtained values are shown in Table 5.

According to different authors [11,15,16], electrostatic interactions between the membrane and charged molecules is an important parameter which determines flux decline. Usually, the pH values of effluents from the dyeing industry are between neutral and basic pH [4]. In this pH range, the polyamide NF99 membrane possesses negative charge, and therefore the negatively charged dye molecules (AB83, AR, MO and SY) are not electrostatically attracted towards the membrane, and hence they do not significantly reduce the permeate flux. However, AB83 and MO dyes molecules showed a decrease in permeate flux, being more significant in the case of MO. This behavior could be explained due to other types of interactions, such as hydrophobic ones (between the aromatic rings of both the dyes and the polyamide membrane selective layer) or hydrogen bonds that can play an important role in membrane blocking, especially under the conditions in which the acidic or basic groups in dyes are partially dissociated.

Positively charged dye molecules (CV) of relatively low molecular weight exhibit a strong fouling effect in the neutral as well as the alkaline pH of the feed solution. This behavior is according to the results obtained by Chindambaram et al. [16].

### 3.5. Fouling Phenomenon after Treatment of Dyes Solutions

A simple means of evaluating the fouling phenomenon effect on the membrane is to repeat the distilled water assays after the dye assays are carried out. In this research, the fouling factor of the membrane, %*FF*, was calculated in order to quantify the fouling phenomenon by comparing the initial, *L_p_*_0_, and final, *L_pf_*, values of the permeability coefficient. The equation is the following:

(4) $$\%FF=\frac{(L_{p0}-L_{pf})}{L_{p0}}\cdot 100$$

Table 6 shows the results of the fouling factor of the membrane after the use of the different dyes studied.

Considering the molecular weight and molecular volume values obtained for each dye, it was found that the smaller dye molecules (MO and BF), whose sizes were close to the molecular weight cut-off (MWCO) of the membrane (200 Da), presented a higher fouling factor. This fact showed that these dyes were absorbed in the membrane and, consequently, the fluxes were reduced. Some authors also described adsorption phenomena for SY [15]. When comparing two dyes of similar molecular size (MO and BF, or CV and AB83), the dye molecules with negative charges and of a linear size gave a lower fouling factor that those of positive charges and with flat disc shape. These results were already described in other studies [23,24].

### 3.6. Morphologic Study of the Membrane

Even though there are many available techniques for observing the membrane surface (including the active layer and the sublayer that sustains it), the most employed technique for nanofiltration membrane characterization is Scanning Electron Microscopy (SEM).

In this research, the samples of native membrane and used membrane (after carrying out the assay of the dye of higher molecular weight, AB83) were analyzed.

**Figure 4:**
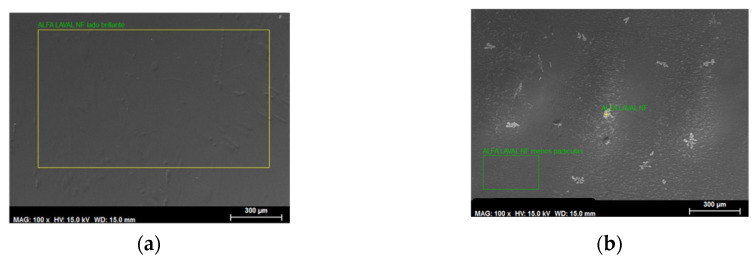
SEM taken picture (300x) of the Alfa Laval NF membrane (**a**) before starting the assays and (**b**) after them.

**Figure 5:**
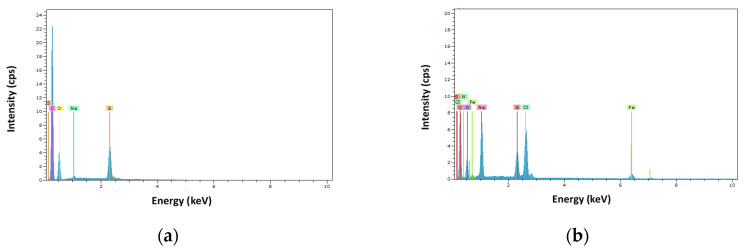
EDX analysis of the membrane (**a**) before the initial assay and (**b**) after the pass of Acid Brown-83 solutions through the membrane.

Figure 4a,b shows an SEM picture (300×) of the membrane Alfa Laval NF before starting the assays and after them. Figure 5 shows the energy-dispersive X-ray spectrum of the membrane (a) before the initial assay and (b) after the pass of Acid Brown-83 solutions through the membrane.

When comparing Figure 4 and Figure 5 for the study of the evolution of the Alfa Laval NF membrane after its use, it can be observed that the SEM picture shows that there is a certain degree of fouling. Membrane fouling is mainly observed on the active layer.

Furthermore, according to the energy-dispersive X-ray spectrum, new elements, such as chlorine, iron and nitrogen appear to be on the membrane surface after the assays. The presence of these elements can be explained because of the pass of Sodium Chloride and the dye solution through the membrane, and because of metallic rests from the installation.

### 3.7. Application of the Spiegler–Kedem–Katchalsky Model

In the bibliography, some adequate models to explain the behavior of the separation process for a thin-layer membrane have been described [25,26,27]—for example, the solution-diffusion model. Therefore, other models are based on the use of coefficients that relate the permeate flux and the fouling factor of the membrane, but in recent years, the most-used models are based on phenomenological transport.

Those models correlate driving force and flow linearly:

(5) $$J_{i}=L_{i,j}\cdot X_{j}$$

where *J_i_* is the flow density of the component, *X_j_* is the driving force and *L_i,j_* is the proportionality coefficient.

The driving forces that dominate the transference of matter in membrane processes are the gradient of pressure and the gradient of concentration.

The Spiegler–Kedem–Katchalsky model [28,29] expresses the initial equations of the previous model in a differential way; not linearly. As a result, it considers that the densities of flux vary through the thickness of the membrane.

(6) $$J_{v}=L_{p}\cdot \left(\frac{dP}{dx}-\sigma \frac{d\Pi }{dx}\right)$$

(7) $$J_{s}=P_{s}\cdot \frac{dC_{s}}{dx}+(1-\sigma )\cdot C_{s}\cdot J_{v}$$

When expressing both equations incrementally:

(8) $$J_{v}=L_{p}\cdot (\Delta P-\sigma \cdot \Delta \Pi )$$

(9) $$J_{s}=P_{s}\cdot \left(C_{m}-C_{p}\right)+(1-\sigma )\cdot J_{v}\cdot C_{s}$$

The Spiegler–Kedem–Katchalsky model was initially developed for reverse osmosis processes; however, it has been proven that it is also applicable in some nanofiltration processes [30,31].

This model assumes that transport coefficients are independent of solute concentration. Nevertheless, these coefficients depend on solute concentration for ionic solutions in nanofiltration membranes. As a result, some authors made some changes in the model to consider this fact [32].

There are two parameters to be determined for the Spiegler–Kedem–Katchalsky model:

- Reflection coefficient (σ). This indicates the capacity of the membrane to be permeated by the solute. A σ = 0 value indicates that the membrane is completely permeable for the solute, whereas a σ = 1 value indicates that the solute is unable to go through the membrane, as it is completely impermeable (total reflection).
- Solute permeability coefficient (Ps). It is defined as the speed at which the solute passes through the membrane. It is unique for each compound and membrane. It is measured in m/s.

The pass of a solute flux through the membrane is caused by two different fluxes: a convective flux, which is caused by the application of a gradient of pressure through the membrane, and a diffusive flux, which is caused by the gradient of concentration in both sides of the membrane. The reflection coefficient is also an indicator of what type of flux prevails: the closer the σ values are to 1, the lower participation has the convective flux [33].

For ideal reverse osmosis membranes, σ values are close to 1 as they present a dense structure and no pores that would enable convective transport.

The observed rejection was calculated using the following expression:

(10) $$\%R_{obs}=\frac{(1-F)}{1-\sigma \cdot F}\cdot 100$$

where *F* is a parameter that depends on the reflection coefficient, solvent flux, and solute permeability coefficient [34]:

(11) $$F=e^{\left(1-\frac{1-\sigma }{P_{s}}\cdot J_{v}\right)}$$

The transport phenomenon through the membrane is, in fact, a combination of convection, solution, and diffusion. In this case, the transport process can be described as an irreversible thermodynamic phenomenon. The following relations among the parameters of the process: reflection coefficient and solute permeability (*σ* and *P_s_*), solvent flux (*J_v_*) and observed rejection (*R_obs_*) were proposed by Spiegler, Kedem and Katchalsky:

(12) $$Ln\left[X\right]=\;1-\frac{1-\sigma }{P_{s}}\;\cdot J_{v}$$

(13) $$X=\;\left(\frac{1}{\left(1-\sigma \right)}-\;\frac{1}{1-R_{obs}}\right)\cdot \frac{\left(1-\sigma \right)}{\sigma }$$

The parameters of the model were obtained by employing both Equations (12) and (13) along with (9). When combining Equations (12) and (13), Equation (14) is obtained:

(14) $$Ln\;\left[\left(\frac{1}{\left(1-\sigma \right)}-\;\frac{1}{1-R_{obs}}\right)\cdot \frac{\left(1-\sigma \right)}{\sigma }\right]=1-\frac{1-\sigma }{P_{s}}\;\cdot J_{v}$$

The average *R_obs_* value was calculated from the experimental data of rejection coefficients; thus it is now a known value. From this value, a parameter z ($\frac{1}{1-R_{obs}}$) was calculated.

Equations (9) and (14) were employed to determine the rejection coefficient (*σ*) and the permeability coefficient (*Ps*). It was determined that solute concentration in the feed was the same as the solute concentration in the membrane (*C_m_* ≈ *C*_0_), as few polarization processes occur. The analyzed solute feeding and permeate concentrations are converted to mol/m^3^ by dividing by the molecular weight of the different dyes.

When replacing *J_s_*, *J_v_*, *C*_0_ and *C_p_* in Equation (9), and after isolating *P_s_*, the following value, dependent on *σ*, is obtained:

(15) $$P_{s}=\;\frac{J_{s}\;-J_{v}\cdot C_{s}\cdot (1-\sigma )}{C_{0}-C_{p}}$$

This would lead to a *P_s_* = *a* − *b*·(1 − *σ*) type of equation, so Equation (14) would become the following:

(16) $$Ln\;\left[\left(\frac{1}{\left(1-\sigma \right)}-\;z\right)\cdot \frac{\left(1-\sigma \right)}{\sigma }\right]-1+\frac{1-\sigma }{a-b\cdot \left(1-\sigma \right)}\;\cdot J_{v}=0$$

where $a=\frac{J_{s}}{C_{0}-C_{p}}$ and $b=\frac{J_{v}\cdot C_{s}}{C_{0}-C_{p}}$.

In order to solve this equation of one unknown parameter (*σ*), it is necessary to use a numeric method, since there is no analytical solution. The program Solver from Excel was employed for that purpose. As a result, the parameters σ and *Ps* were obtained for each different case. The results are shown in Table 7.

**Figure 6:**
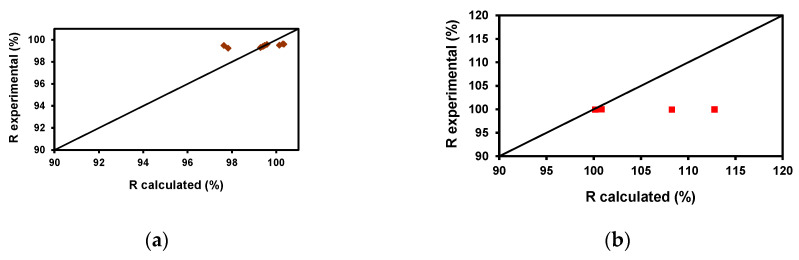

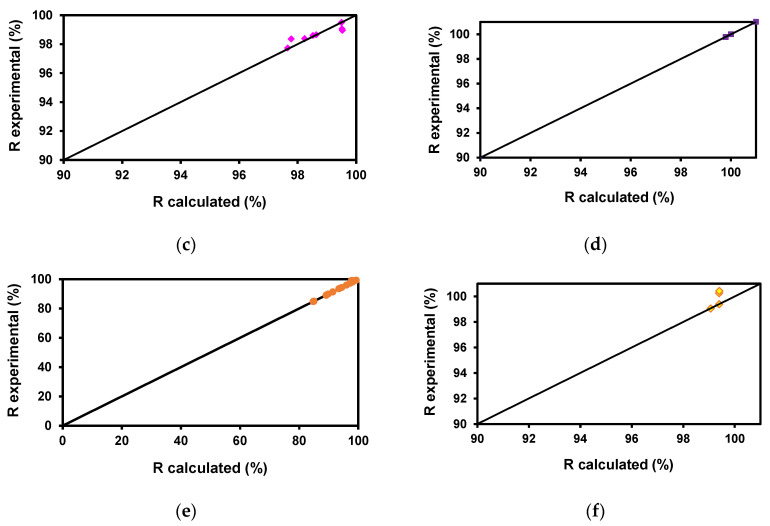
Correlation between the experimental values of the rejection coefficient and those calculated by the model. (**a**) ♦ AB83, (**b**) ■ AR, (**c**) ♦ BF, (**d**) ■ CV, (**e**) ● MO, (**f**) ◊ SY.

To verify the model, the values of F and Robs were calculated. The following figures (Figure 6a–f) show the good correlation in most cases between the experimental values of the rejection coefficient and those calculated by the model. Table 5 shows the standard deviation values being the highest lower than 4%.

### 3.8. Comparative Study of the Results

A comparison of the results obtained on permeate flux and rejection coefficient using NF99 for the different dyes molecules was carried out. Table S1 (Supplementary Material) shows the results obtained by other authors using other membranes (native and modified) for the removal of dyes.

## 4. Conclusions

The performance of a polyamide nanofiltration membrane on the removal of six different dyes, Acid Brown-83, Allura Red, Basic Fuchsin, Crystal Violet, Methyl Orange and Sunset Yellow, has been studied. Firstly, the membrane characterization was carried out, obtaining a water permeability coefficient value of 1.665 × 10^−8^ s m^−1^. The membrane selectivity was also determined, and the solute permeability coefficients were 6.705 × 10^−6^ and 1.632 × 10^−7^ for NaCl and MgCl_2_, respectively. It has been proven that the chemical structure of the dyes has an important influence on the permeate fluxes and rejection coefficients obtained, these being the molecular volume and the length perpendicular to the maximum area the most relevant parameters. The pH influence was also studied, these being the membrane negatively charged at neutral and basic pH and therefore being repelled by the dye molecules of negative charge (AB83, AR, MO and SY). However, AB83 and MO dye molecules showed a decrease in permeate flux, which can be explained due to other types of interactions (hydrophobic interactions and the presence of hydrogen bonds that cause membrane blocking). Membrane fouling was determined by calculating a fouling factor, showing that the smaller dye molecules (Methyl Orange and Basic Fuchsin) presented the highest fouling. Additionally, when comparing dyes of similar molecular sizes, those with negative charges and linear size gave lower values of fouling factor. The morphologic study of the membrane by Scanning Electron Microscopy (SEM) and infrared spectrum confirmed the observed degree of fouling. Finally, the Spiegler–Kedem–Katchalsky model that simulates the membrane behavior was successfully applied, with a high degree of agreement between the experimental and calculated rejection coefficients.

## Figures and Tables

**Table 1:** Characteristics of the membrane used in the experimental test module.

**Manufacturer**	Alfa Laval (Denmark)
**Product denomination**	NF99
**Type**	Thin-film composite on polyester
**Composition**	Polyamide
**Membrane surface area (m^2^)**	0.003
**Maximum pressure (N m^−2^)**	55 × 10^5^
**MgSO_4_ rejection (%)** **(2 kg m^−3^, 9·10^5^ N m^−2^)**	≥98
**NaCl rejection (%)**	>90
**pH range**	3−10
**Temperature range (°C)**	5–50

**Table 2:** Chemical properties of some of the dyes employed in the study. Data obtained using PubChem. https://pubchem.ncbi.nlm.nih.gov/.

Dyes	Log K_ow_	pK_a_	Water Solubility (g L^−1^)
**AB83**	-	-	>100
**AR**	−0.55	-	225
**BF**	1.632 (*)	-	1–5
**CV**	0.96 0.51	pK_a1_ = 5.31 pK_a2_ = 8.64	4
**MO**	−0.66 (*)	-	0.2 5.0 (*)
**SY**	−1.18	pK_a1_ = 0.82 pK_a2_ = 1.46	190

(*) https://www.carlroth.com/medias/.

**Table 3:** Properties of the membrane used in the experimental assays.

Membrane	Water Permeability L_p_ (m s^−1^)	Solute Permeability P_s_ (m·s^−1^)	
		NaCl	MgCl_2_
**NF99**	1.665 × 10^−8^	6.705 × 10^−6^	1.632 × 10^−7^
**References** [13,19,20]	1.5 × 10^−8^	-	-

**Table 4:** Structure parameters of the dye molecules. Data obtained using ChemAxon. https://chemaxon.com/products/marvin.

Dyes	Allura Red	Basic Fuchsin	Crystal Violet	Methyl Orange	Sunset Yellow
**Dreiding energy** **(kcal/mol)**	318.39	186.09	294.85	237.39	311.43
**MMFF94 energy** **(kcal/mol)**	196.18	69.17	121.74	93.12	184.7
**Minimal projection area** **(Å^2^)**	51.15	55.59	71.18	30	46.88
**Maximal projection area (Å^2^)**	117.52	81.35	105.67	93.1	105.50
**Minimal projection radius (Å)**	5.33	6.28	7.49	3.96	5.78
**Maximal projection radius (Å)**	8.57	6.76	8.06	8.58	8.40
**Length perpendicular to the max area (Å)**	8.44	7.46	8.24	5.88	9.43
**Length perpendicular to the min area (Å)**	16.78	12.60	14.84	17.33	16.22
**Van der Waals volume** **(Å^3^)**	338.50	270.57	378.31	254.85	295.56

**Table 5:** Rejection coefficient and permeate flux variation with pH of feed for the different dyes. P = 15 bar; [Dye] = 50 mg/L.

Dyes	Permeate Flux (kg/m^2^ s) × 10^3^			Rejection Coefficients (%)		
	pH = 3	pH = 7	pH = 8	pH = 3	pH = 7	pH = 8
**AB83**	35.44	33.50	33.33	99.40	99.48	99.56
**AR**	39.20	41.11	38.40	99.99	99.93	99.95
**BF**	-	30.86	-	-	98.78	-
**CV**	38.66	32.96	16.75	99.91	99.78	99.98
**MO**	37.87	31.72	19.17	97.27	87.47	99.02
**SY**	34.67	40.00	38.67	99.78	99.22	99.88

**Table 6:** Values of fouling factor of the membrane for the different dye assays.

Dyes	AB83	AR	BF	CV	MO	SY
**FF (%)**	10.65	0.6	87.23	24.13	35.29	3.44

**Table 7:** Solute permeability coefficient and reflection parameter for the different dyes obtained using SKK model.

Dyes	*P_s_* (m s^−1^)	*σ*	Standard Deviation
**AB83**	1.6418 × 10^−7^	0.9954	0.3605
**AR**	2.6624 × 10^−8^	0.9994	3.8356
**BF**	2.021 × 10^−7^	0.9887	0.1692
**CV**	1.0198 × 10^−7^	0.9974	1.0023
**MO**	7.8114 × 10^−7^	0.9563	0.2147
**SY**	2.5221 × 10^−7^	0.9942	0.3382

## Supplementary Materials

The following are available online at https://www.mdpi.com/2077-0375/10/12/408/s1, Figure S1: Rejection coefficient (♦) and permeate flux (■) variation with dreiding energy (A&B) and with MMFF94 Energy (C&D) for colorants: (MO) Methyl Orange, (BF) Basic Fuchsin, (SY) Sunset Yellow, (AR) Allure Red, (CV) Crystal Violet. Experimental conditions: pH = 7, [Dyes] = 50 mg/L and pressure 10 bar (A&C) and 15 bar (B&D). Figure S2: Rejection coefficient (♦) and permeate flux (■) variation with minimal projection area (A&B) and with maximal projection area (C&D) for colorants: (MO) Methyl Orange, (BF) Basic Fuchsin, (SY) Sunset Yellow, (AR) Allure Red, (CV) Crystal Violet. Experimental conditions: pH = 7, [Dyes] = 50 mg/L and pressure 10 bar (A&C) and 15 bar (B&D). Figure S3: Rejection coefficient (♦) and permeate flux (■) variation with minimal projection radius (A&B) and with maximal projection radius (C&D) for colorants: (MO) Methyl Orange, (BF) Basic Fuchsin, (SY) Sunset Yellow, (AR) Allure Red, (CV) Crystal Violet. Experimental conditions: pH = 7, [Dyes] = 50 mg/L and pressure 10 bar (A&C) and 15 bar (B&D). Figure S4: Rejection coefficient (♦) and permeate flux (■) variation with length perpendicular to the minimal area (A&B) and with molecular weight (C&D) for colorants: (MO) Methyl Orange, (BF) Basic Fuchsin, (SY) Sunset Yellow, (AR) Allure Red, (CV) Crystal Violet. Experimental conditions: pH = 7, [Dyes] = 50 mg/L and pressure 10 bar (A&C) and 15 bar (B&D), Table S1: Comparison of dye removal between previous studies and this study in terms of water flux and rejection.

## Nomenclature

*C*_0_	solute concentration in the feed (kg/m^3^)
*C_m_*	solute concentration in the membrane (kg/m^3^)
*C_p_*	solute concentration in the permeate (kg/m^3^)
*C_s_*	logarithmic average solute concentration between feeding and permeate (kg/m^3^)
*FF*	fouling factor (%)
*J_s_*	solute flux density (kg/m^2^s)
*J_v_*	solvent flux density (kg/m^2^s)
*L_p_*	solvent permeability coefficient (m/s)
*L*_*p*0_	initial solvent permeability coefficient (m/s)
*L_pf_*	final solvent permeability coefficient (m/s)
*P_s_*	solute permeability coefficient (m/s)
*r*	rejection (dimensionless)
*R*	rejection coefficient (%)
*R_obs_*	observed rejection coefficient (%)
Δ*P*	operating pressure (P_a_)
Δ*П*	osmotic pressure (P_a_)
*σ*	reflection coefficient
