# Supplementary material for: Removal of Different Dye Solutions: A Comparison Study Using a Polyamide NF Membrane
Source: Membranes (Basel). 2020 Dec 10;10(12):408. doi: 10.3390/membranes10120408 (PMC7764484; doi:10.3390/membranes10120408)
Supplement: Supplementary file 1 [file membranes-10-00408-s001.pdf]

Supporting Information

Removal of Different Dye Solutions: A Comparison Study Using a Polyamide NF Membrane

Asunción María. Hidalgo [1,*], Gerardo León [2], María Gómez [1], María Dolores Murcia [1], Elisa Gómez [1] and José Antonio Macario [1]

[1] Departamento de Ingeniería Química, Facultad de Química, Campus de Espinardo, Universidad de Murcia, 30100 Murcia, Spain; maría.gomez@um.es (M.G.); md.murcia@um.es (M.D.M.); egomez@um.es (E.G.); joseantonio.macario@um.es (J.A.M.)

[2] Departamento de Ingeniería Química y Ambiental, Universidad Politécnica de Cartagena, 30202 Cartagena, Spain; gerardo.leon@upct.es

* Correspondence: ahidalgo@um.es; Tel.: +34-868-887-353

Table S1. Comparison of dye removal between previous studies and this study in terms of water flux and rejection.

Membrane	Target Dye	Experimental conditions	Water Flux (L/m^2 h bar)	Rejection (%)	Reference
**NF99**	Acid Brown-83	50 mg/L dye at 10 bar	66.6	99.36	This work
**NF99**	Allura Red	50 mg/L dye at 10 bar	82.9	99.92	This work
**NF99**	Basic Fuchsin	50 mg/L dye at 10 bar	79.0	93.66	This work
**NF99**	Crystal Violet	50 mg/L dye at 10 bar	72.9	99.77	This work
**IM26**	Crystal Violet	100 mg/L dye at 4 bar	30.8	88.4	Cheng et al. 2016
**Psf UF**	Crystal Violet	100 mg/L dye at 0.8 bar	38.2	46.8	Cheng et al. 2016
**EGCG-PEI-TC/PTFE**	Crystal Violet	100 mg/L dye at 2 bar	4	100	Zhang et al. 2020
**Modified Psf (M1)**	Crystal Violet	100 mg/L dye at 4.14 bar	2.2	85	Rambabu et al. 2019
**NF99**	Methyl Orange	50 mg/L dye at 10 bar	78.5	88.49	This work
**EGCG-PEI-TC/PTFE**	Methyl Orange	100 mg/L dye at 2 bar	5.5	100	Zhang et al. 2020
**ANF TFC**	Methyl Orange	100 mg/L dye at 4 bar	8.4	98.6	Li et al. 2019
**PMIA TFC**	Methyl Orange	100 mg/L dye at 4 bar	1.2	100	Li et al. 2019
**NF90**	Methyl Orange	100 mg/L dye at 4 bar	4	100	Li et al. 2019
**NF 270**	Methyl Orange	100 mg/L dye at 4 bar	9.3	98.6	Li et al. 2019

**PS/PEI (20%)**	Methyl Orange	50 mg/L dye at 4 bar	16.30	64.7	Benkhaya et al. 2020
**NF99**	Sunset Yellow	50 mg/L dye at 10 bar	86.4	99.39	This work
**NF 90**	Sunset Yellow	50 mg/L dye at 5 bar	14.9	>99	Jun et al. 2019
**Treat pH 13.5 ,7D**	Sunset Yellow	50 mg/L dye at 5 bar	16.0	>99	Jun et al. 2019
**DEA-modified PA-TFC**	Sunset Yellow	100 mg/L dye at 5 bar	14.8	97.5	Liu et al. 2017
**PA-TFC**	Sunset Yellow	100 mg/L dye at 5 bar	9.3	>99	Lü et al. 2019
**CMCNa/PP composite membrane**	Sunset Yellow	100 mg/L dye at 5 bar	8.6	82.2	Yu et al. 2012
**Sericin-TCM**	Sunset Yellow	100 mg/L dye at 5 bar	12.4	95.4	Zhou et al. 2014

References

Benkhaya, S.; M′rabet, S.; Hsissou, R.; El Harfi, A. Synthesis of new low-cost organic ultrafiltration membrane made from Polysulfone/Polyetherimide blends and its application from soluble azoic dyes removal. *J. Mater. Res. Technol*. **2020**, *9*, 4763–4772.

Cheng, L.; Zhu, L.P.; Zhang, P.B.; Sun, J.; Zhu, B.K.; Xu, Y.Y. Molecular separation by poly (N-vinyl imidazole) gel-filled membranes. *J. Membrane Sci*. **2016**, *497*, 472–484.

Jun, B.M.; Yoon, Y.; Park, C.M. Post-treatment of nanofiltration polyamide membrane through alkali-catalyzed hydrolysis to treat dyes in model wastewater. *Water*. **2019**, *11*, 1645–1659.

Li, Y.; Wong, E.; Mai, Z.; Der Bruggen, B.V. Fabrication of composite polyamide/Kevlar aramid nanofiber nanofiltration membranes with high permselectivity in water desalination. *J. Membrane Sci*. **2019**, *592*, 117396–117407.

Liu, M.; Chen, Q.; Lu, K.; Huang, W.; Lü, Z., S.; Zhou, C.; Yu, S.; Gao, C. High efficient removal dyes from aqueous solution through nanofiltration using diethanolamine-modified polyamide thin-film composite membrane. *Sep. Purif. Technol.* **2017**, *137*, 135–143.

Lü, Z.; Hu, F.; Li, H.; Zhang, X.; Yu, S.; Liu, M. Gao, C. Composite nanofiltration membrane with asymmetric selective separation layer for enhanced separation efficiency to anionic dye aqueous solution. . *J. Hazard. Mater.* **2019**, *368*, 436-443.

Rambabu, K.; Bharath, G.; Monash, P.; Velu, S., Banat, F.; Naushad, M.; Arthanareeswaran, G.; Show, P.L. Effective treatment of dye pollutant wastewater using nanoporous CaCl2 modified polyethersulfone membrane. *Process Safety Environ. Protection*, **2019**, *124*, 266-278.

Yu, S.; Chen, Z.; Cheng, Q.; Lü, Z.; Liu, M. Gao, C. Application of thin-film composite hollow fiber membrane to submerged nanofiltration of anionic dye aqueous solutions. *Sep. Purif. Technol*. **2012**, *88*, 121–129.

Zhang, N.; Huang, Z.; Yang, N.; Zhang, L.; Jiang, B.; Sun, Y.; Ma, J. Nanofiltration membrane via EGCG-PEI co-deposition followed by crosslinking on microporous PTFE substrates for desalination. *Sep. Purif. Technol*. **2020**, *232*, 115964–115974.

Zhou, C.; Shi, Y.; Sun, C.; Yu, S.; Liu, M; Gao, C. Thin-film composite membranes formed by interfacial polymerization with natural material sericin and trimesoyl chloride for nanofiltration. *J. Membrane Sci*. **2014**, *471*, 381-391.

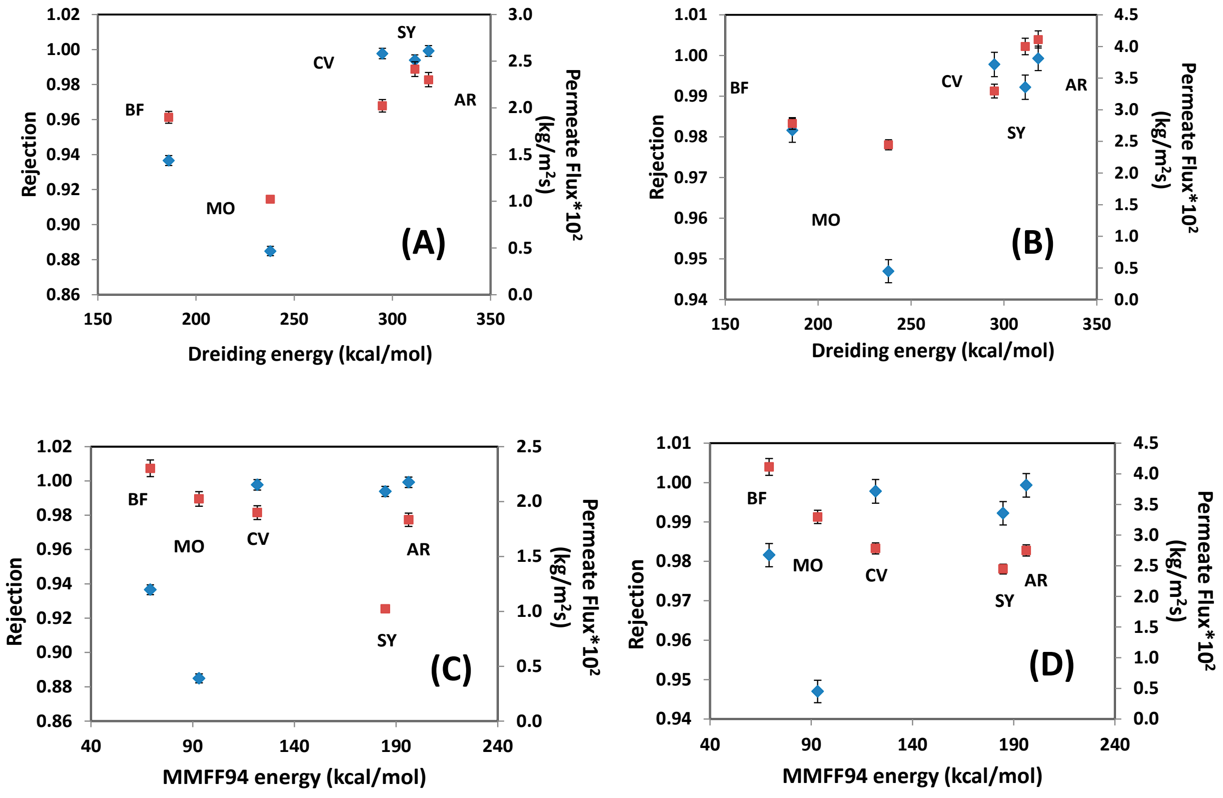

Figure S1. Rejection coefficient (♦) and permeate flux (■) variation with dreiding energy (A&B) and with MMFF94 Energy (C&D) for colorants: (MO) Methyl Orange, (BF) Basic Fuchsin, (SY) Sunset Yellow, (AR) Allure Red, (CV) Crystal Violet. Experimental conditions: pH =7, [Dyes] = 50 mg/L and pressure 10 bar (A&C) and 15 bar (B&D).

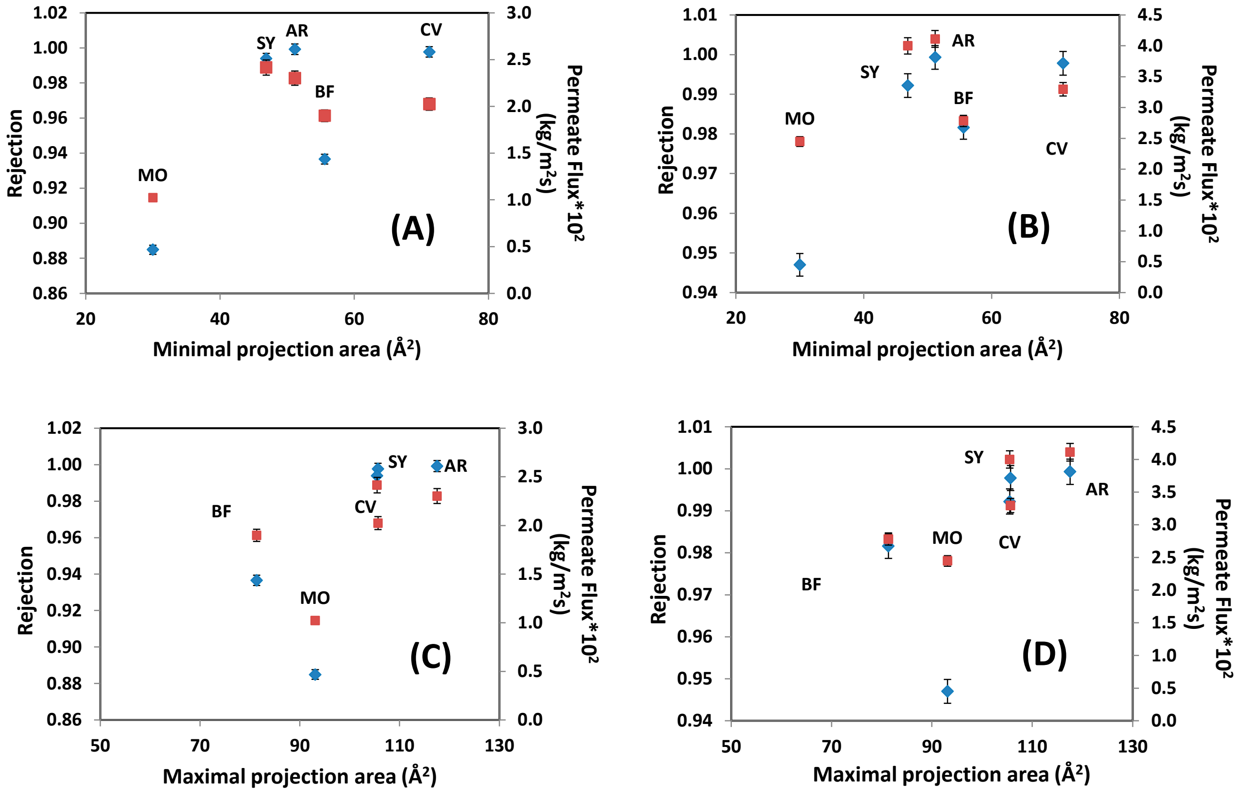

Figure S2. Rejection coefficient (♦) and permeate flux (■) variation with minimal projection area (A&B) and with maximal projection area (C&D) for colorants: (MO) Methyl Orange, (BF) Basic Fuchsin, (SY) Sunset Yellow, (AR) Allure Red, (CV) Crystal Violet. Experimental conditions: pH =7, [Dyes] = 50 mg/L and pressure 10 bar (A&C) and 15 bar (B&D).

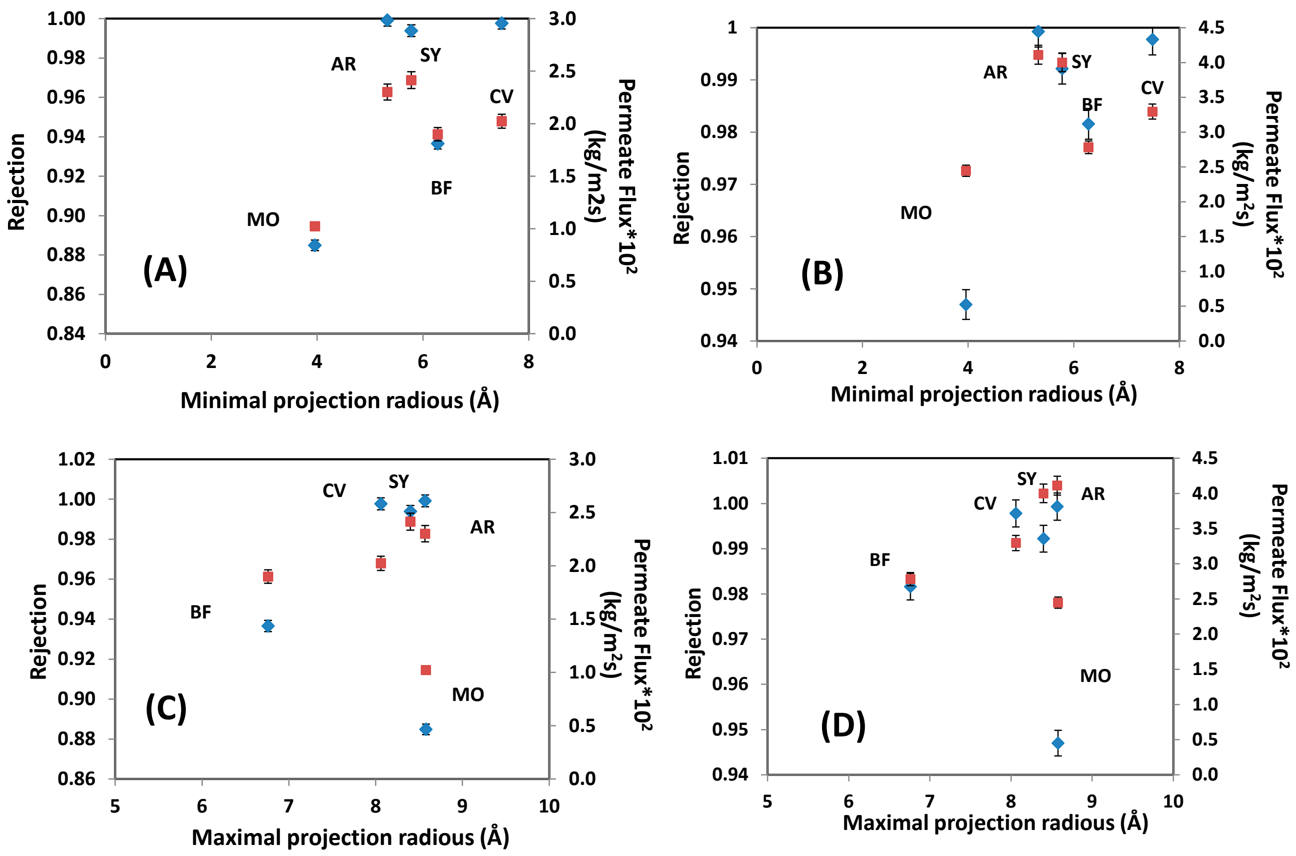

Figure S3. Rejection coefficient (♦) and permeate flux (■) variation with minimal projection radious (A&B) and with maximal projection radious (C&D) for colorants: (MO) Methyl Orange, (BF) Basic Fuchsin, (SY) Sunset Yellow, (AR) Allure Red, (CV) Crystal Violet. Experimental conditions: pH =7, [Dyes] = 50 mg/L and pressure 10 bar (A&C) and 15 bar (B&D).

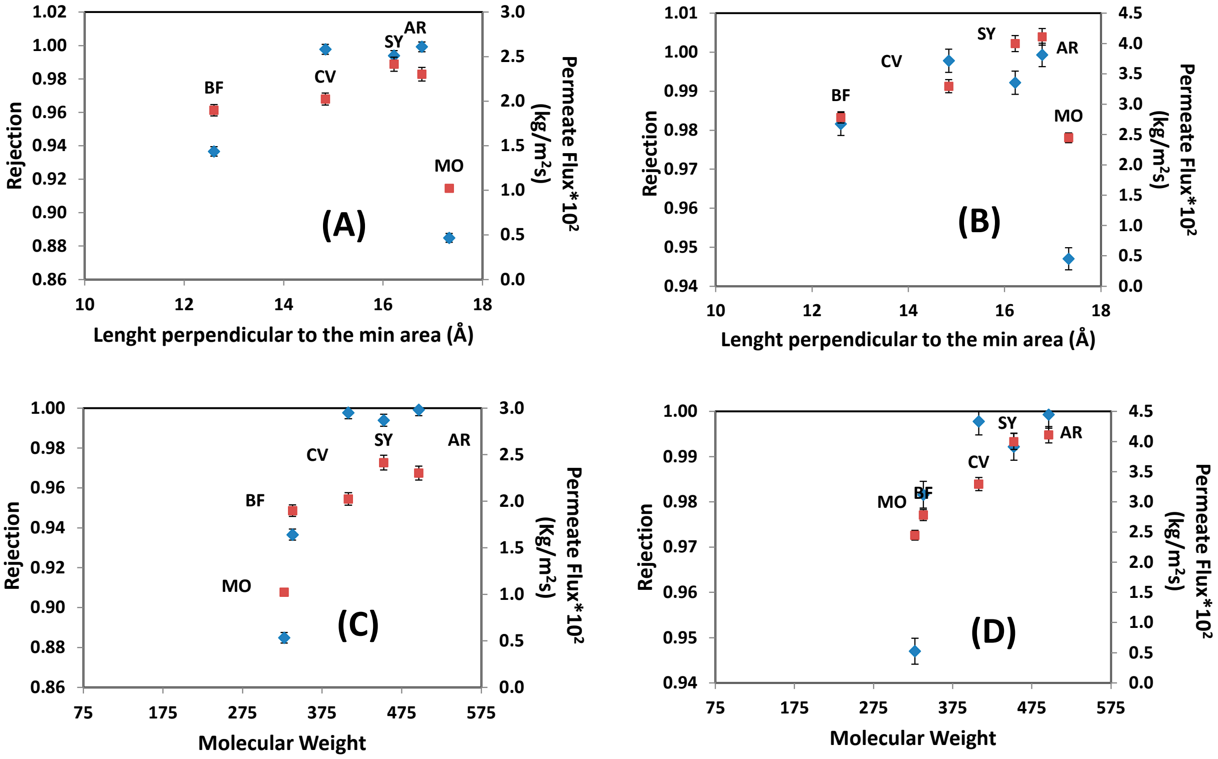

Figure S4. Rejection coefficient (♦) and permeate flux (■) variation with length perpendicular to the minimal area (A&B) and with molecular weight (C&D) for colorants: (MO) Methyl Orange, (BF) Basic Fuchsin, (SY) Sunset Yellow, (AR) Allure Red, (CV) Crystal Violet. Experimental conditions: pH =7, [Dyes] = 50 mg/L and pressure 10 bar (A&C) and 15 bar (B&D).
